# Beyond cervicovaginal limitations: potential bidirectional interactions between HPV infection and mucosal microbiota from oral, anorectal and penile sites — a mini-review

**DOI:** 10.3389/fcimb.2026.1962165

**Published:** 2026-09-22

**Authors:** Wei Liu, Qiuyang Yu, Xiao Xue

**Affiliations:** 1 Department of Dermatology and Venereology, First Hospital of Jilin University, Changchun, China; 2 Department of Laboratory Medicine, First Hospital of Jilin University, Changchun, China

**Keywords:** human papillomavirus, mucosal microbiota, sexually transmitted infection, skin Infection, skin microbiome

## Abstract

Most existing studies focusing on human papillomavirus (HPV)-microbiota interactions are confined to the cervicovaginal tract, while mucosal microecological crosstalk at oral, anorectal and penile sites remains insufficiently summarized. This mini-review provides a narrative synthesis of current sequencing and epidemiological evidence regarding potential bidirectional interactions between HPV infection and site-specific mucosal microbiota across extra-cervicovaginal niches in both males and females. Oral HPV reshapes microbial composition in a sex-, race- and population-dependent manner; periodontal dysbiosis, high-risk HPV and *Candida albicans* jointly form a tripartite oncogenic axis that may contribute to oral squamous cell carcinoma, and periodontal inflammation independently elevates oral HPV susceptibility. Among men who have sex with men, pro-inflammatory anaerobes such as *Bacteroides fragilis* and *Sneathia* accumulate in anorectal HPV-related lesions, and concurrent HIV or sexually transmitted bacterial co-infections worsen mucosal dysbiosis and viral persistence. On penile skin, *Corynebacterium* is associated with a lower prevalence of HPV, whereas anaerobe-dominant community state types increase high-risk HPV risk, with HIV-induced baseline dysbiosis masking HPV-specific penile microbial signatures. Collectively, tissue-specific mucosal dysbiosis may represent an important co-factor linked to persistent HPV infection and subsequent malignant transformation. This review offsets long-standing sex-related and anatomical research bias, clarifies multi-site host-virus-microbe mechanisms, and provides theoretical support for developing microbiota-based biomarkers and microecological interventions to prevent non-cervical HPV-associated malignancies.

## 1 Introduction

Human Papillomavirus (HPV) is the most common sexually transmitted infection globally, with the most sexually active men and women estimated to have been infected with genital HPV at least once during their lifetime (Bruni et al., 2023). HPV can be transmitted in many ways through different abrasions, with sexual intercourse, when the newborn passes through an infected genital tract, and from a variety of self-inoculation positions (Erickson et al., 2013; Muzio et al., 2021). HPVs are capable of infecting multiple human anatomical niches including the skin, vagina, cervix, oral cavity and gut. Distinct dominant high-abundance HPV genotypes are enriched at separate body sites, exhibiting prominent tissue-specific tropism (Ma et al., 2014).

The mechanisms underlying persistent HPV infection remain incompletely elucidated, with mucosal microbial dysbiosis emerging as an important contributing factor. Studies have shown that microbial imbalance is associated with sustained HPV inflammation and may contribute to the initiation and progression of high-grade squamous intraepithelial lesions (HSIL) (Song et al., 2020; Lebeau et al., 2022). Certain bacterial metabolites can activate epithelial inflammasomes, amplify inflammatory cascades, and potentiate the activity of HPV oncoproteins, thereby reinforcing persistent viral infection (Song et al., 2020; Yang et al., 2025). Each individual harbors a unique microbial fingerprint. Organ-specific microbiotas regulate host immunity and mucosal physiological functions (Kilian et al., 2016; Zhang et al., 2022).

Extensive research has delineated the complicated interplay between cervicovaginal microbiota and HPV, confirming its pivotal role in cervical carcinogenesis (Usyk et al., 2020; Huang et al., 2024; Oyedokun et al., 2026). However, bidirectional crosstalk between HPV and mucosal flora at oral, anorectal and penile sites remains insufficiently characterized, and few reviews have provided a summary of these extra-cervicovaginal microbial niches. This narrative mini-review integrates available evidence for extra-cervicovaginal mucosal niches in both sexes to mitigate the historical cervix-centered research bias (**Table 1**). Elucidating the correlation between extra-cervicovaginal microbiomes and HPV infection will further clarify mucosal HPV susceptibility and relevant host-microbe-viral interactions.

**Table 1:** Summary of representative included studies investigating HPV-microbiota associations across oral, anorectal and penile mucosal sites.

Anatomical site	Study population	Sample size	Study design	Specimen type	Sequencing/method	Main findings	References
Oral	US adults from nationally representative NHANES	N=5496	Cross-sectional	Oral rinse	16S rRNA (V4 region), QIIME; multivariable logistic regression	Reduced oral α-diversity was independently associated with high-risk oral HPV only among males. Significant β-diversity differences by high-risk HPV status were also restricted to males.	Feng et al. (2024)
Oral	Adult participants; stratified by periodontitis status	N=4685	Cross-sectional	Oral rinse	16S rRNA amplicon sequencing; adjusted logistic regression	Moderate-to-severe periodontitis was associated with higher odds of oral HPV infection only in females.	Liang et al. (2026)
Oral	Pregnant women (third trimester)	N=39	Cross-sectional	Maternal oral swabs	16S rRNA V3-V4 amplicon sequencing; QIIME, LEfSe	Higher bacterial richness in HPV-positive maternal oral samples. *Selenomonas* spp., *Megasphaera* and TM7 taxa enriched in HPV-positive oral specimens.	Tuominen et al (2018a)
Oral	Risk-enrolled adult participants from MOUTH study*	N=495	Cross-sectional	Saliva; oral-rinse	16S rRNA V4 amplicon sequencing; PICRUSt2; Corncob differential abundance analysis	Significant shifts in β-diversity were observed. HPV-positive subjects had higher abundance of Actinomycetaceae, Prevotellaceae, Veillonellaceae, Campylobacteraceae and Bacteroidete.	Zhang et al. (2022)
Oral	Adults with non-cancer oral HPV status	N=127	Cross-sectional (re-analysis of publicly available data)	Oral swab	Full-length 16S rRNA sequencing; ecotype clustering; VFDB-based inferred virulence-ecology profiling	Oral HPV did not drive discrete oral-microbiome taxa shifts or define microbiome ecotypes. HPV-positivity aligned with pre-existing high-virulence-pressure oral ecological landscapes rather than reshaping community composition.	Chambuso et al. (2026)
Oral	US adults from NHANES 2009-2012	N=5534	Cross-sectional	Oral rinse	16S rRNA V4 amplicon sequencing; LDA topic-modelling, ADAPT differential-abundance analysis	Higher α-diversity was observed in HPV-positive Non-Hispanic Black participants. *Sneathia* and *Odoribacterium* were enriched among HPV-positive individual.	Byrd et al. (2026)
Oral	HIV-negative and HIV-positive MSM	N=63	Cross-sectional	Oral swabs	16S rRNA amplicon sequencing	HrHPV-linked oral microbial alterations were only detectable in HIV-negative MSM. Pre-existing HIV-associated oral dysbiosis may mask HPV-specific microbial signature.	Escobar-Marcillo et al. (2025)
Oral	Healthy Australian adults (stratified by HPV-negative, low-risk HPV, high-risk HPV status)	N=127	Cross-sectional retrospective analysis	Saliva	PacBio HiFi full-length 16S rRNA gene sequencing; QIIME2, DESeq2	HPV-negative males harboured higher *Streptococcus salivarius*; lr-HPV-positive males showed depletion of *S. salivarius* and *S. parasanguinis*. HPV-positive females had significantly lower Shannon α-diversity and distinct oral β-diversity.	Maidment et al. (2025)
Oral	Breastfeeding mothers and their infants (mother-infant pairs)	N=35	Cross-sectional	Breast-milk; infant oral swabs	16S rRNA V3-V4 amplicon sequencing; QIIME, LEfSe	Infant oral HPV infection altered bacterial community clustering without changing overall α-diversity. *Ureaplasma*, *Lysinibacillus* enriched in HPV-positive infants; *Veillonella dispar* enriched in HPV-negative infants.	Tuominen et al. (2018b)
Anorectal	HIV-negative MSM	N=58	Retrospective analysis based on prospective cohort follow-up	Rectal swabs	16S rRNA (V3-V4 region), QIIME2 pipeline; Wilcoxon test, Metastats, LEfSe	*Bacteroides fragilis* was significantly enriched in participants with persistent anal HPV. *Victivallis*, *Negativibacillus*, *Eggerthella* were enriched in persistent-HPV subjects; *Prevotella disiens* and *Gardnerella* were more abundant in HPV-negative participant.	Yang et al. (2025)
Anorectal	Nigerian MSM	N=113	Cross-sectional pilot study	Anal swabs	16S rRNA (V3-V4 region, Illumina MiSeq); K-means clustering, LEfSe, Fisher’s exact test	Four anorectal microbial community clusters were identified. The *Sneathia*-enriched cluster had the highest proportion of HPV-16-positive participants; most subjects in this cluster were ART-treated, virally-suppressed MSM.	Nowak et al. (2019)
Anorectal	Women with high-grade lower-genital-tract lesions	N=71	Retrospective analysis	Anorectal swabs	16S rRNA V4 amplicon sequencing (Illumina MiSeq), QIIME2, LEfSe, UniFrac-based PCoA	Pro-inflammatory anaerobes including *Peptoniphilus*, *Prevotella*, *Porphyromonas* and *Fusobacterium* were significantly enriched in anal-cancer specimens.	Elnaggar et al. (2023)
Anorectal	STI-clinic-attending MSM	N=92	Cross-sectional	Anorectal swabs	16S rRNA (V3-V4 region, Illumina MiSeq); QIIME pipeline; point-biserial correlation	Hr-HPV positivity was positively correlated with *Escherichia* and *Corynebacterium*, and negatively correlated with multiple Firmicutes taxa including *Prevotella*, *Oscillospira*, *Sutterella*. Elevated α-diversity was observed in CT/NG-infected HPV-positive subjects.	Ceccarani et al. (2024)
Penile	Heterosexually-active Black South African adult men	N=238	Cross-sectional retrospective analysis	Swabs of penile shaft, glans and coronal sulcus	16S rRNA (V3-V4 region), Illumina MiSeq; LEfSe for differential taxa analysis	Six penile CSTs were identified. *Corynebacterium*-dominated CST-1 correlated with lower hr-HPV risk, whereas anaerobe-rich CST-5 was associated with higher HPV/hr-HPV prevalence. Enrichment of *Prevotella*, *Peptoniphilus*, *Dialister* and depletion of *Corynebacterium* were observed in hr-HPV-positive men.	Onywera et al (2020a)
Penile	Sexually-active HIV-negative men	N=103	Cross-sectional	Penile skin swabs	16S rRNA V4-region amplicon sequencing; hierarchical clustering for CSTs	Subclinical HPV infection in HIV-negative men was associated with a dysbiotic PSM, characterized by an increased abundance of anaerobic bacteria alongside with a reduced proportion of facultative anaerobic genera.	Garza-González et al. (2026)

MSM, men who have sex with men; hr-HPV, high risk HPV; lr-HPV, low risk HPV; CST, Community State Type.

*MOUTH study, The Men and Women Offering Understanding of Throat HPV study.

## 2 Oral mucosal microbiota and HPV infection

The mouth is the second largest and diverse microbiota niche after the gut, harboring over 700 species of bacteria (Muzio et al., 2021; Benjamin et al., 2023). The overall prevalence of oral HPV among healthy general populations ranged from 0.67% to 11.89%, and infection rates were higher in males than females (Maltseva et al., 2025). Chronic inflammation linked to the oral microbiome may induce abnormal immune responses and interfere with the clearance of oral HPV infection, which could potentially contribute to carcinogenesis (Tezal, 2012). HPV infection is a significant risk factor for oropharyngeal cancer (McQuillan et al., 2017). Approximately 70% of oropharyngeal cancer are associated with oral HPV infection (D'Souza et al., 2007; Marur et al., 2010; Joseph and D'Souza, 2012). The oral microbiome can also cause periodontal diseases, which are risk factors for both oral HPV infection and head and neck squamous cell carcinoma (Feng et al., 2024). Observational data suggest that members of the oral microbiota may interact with HPV by modifying its pathogenicity or bacterial community behavior (Ghannoum et al., 2010; Muzio et al., 2021).

### 2.1 HPV–oral microbiota interactions in general populations

Two independent population-based oral microbiome studies reported sex-specific microbial alterations correlated with oral HPV infection (Feng et al., 2024; Maidment et al., 2025). One cohort found depleted *Streptococcus salivarius* and *S. parasanguinis* in low-risk HPV-positive males, while HPV-positive females showed lower Shannon α-diversity and distinct oral β-diversity (Maidment et al., 2025). Large-scale NHANES cross-sectional data (n=5496) further confirmed that reduced oral microbial α-diversity and unique community profiles were only significantly associated with high-risk oral HPV among males, a sex-based discrepancy consistent with men’s higher susceptibility to HPV-related head and neck malignancies (Park et al., 2022; Gaeta et al., 2023; Feng et al., 2024).Another cross-sectional study analyzing more than 5000 oral-rinse specimens reported higher relative abundance of Veillonellaceae and reduced *Gemella* among HPV-positive individuals. Notably, *Gemella* was enriched in HPV-associated oropharyngeal cancer, indicating that *Gemella* overgrowth arises in late tumor-related dysbiosis rather than during early-stage HPV infection (Byrd et al., 2026).

Study has confirmed that oral HPV infection reshapes overall oral microbial community β-diversity and distinguished taxonomic features differing by HPV status (Zhang et al., 2022). HPV carriers show enriched Actinomycetaceae, Prevotellaceae, Veillonellaceae and *Campylobacteraceae*, whereas Gemellaceae decline and Bacteroidetes rise in infected individuals (Zhang et al., 2022). Two mechanistic pathways may explain this association. First, periodontopathic *Prevotella* and *Actinomyces* sustain mucosal inflammation and barrier damage to boost HPV entry, echoing epidemiological ties between periodontal disease and HPV-associated head and neck cancer (Tezal et al., 2012; Bui et al., 2013; Cekici et al., 2014; Larsen, 2017). Second, HPV-positive oral microbiota displays impaired xenobiotic degradation of naphthalene, styrene and chloroalkanes; accumulated toxins induce oxidative DNA damage and activate ATM kinase to support HPV replication. Pathogenic *Campylobacter* further releases genotoxins to trigger genomic instability. In summary, metabolically disrupted oral dysbiosis may represent a co-factor associated with increased risks of oral HPV acquisition and persistent infection.

It is reported that oral HPV does not induce distinct oral microbial taxa or ecotypes, but colonizes pre-existing oral ecosystems with coordinated virulence networks (Chambuso et al., 2026). HPV-positive specimens were enriched in periodontal anaerobes favoring viral persistence and oncogenic synergism, with HPV persistence dependent on rearranged functional modules for adhesion, invasion and immune crosstalk rather than elevated total microbial virulence (Chambuso et al., 2026). Such viral-microbe interactions differ drastically from the hypoxic, immune-suppressed microenvironment of HPV-driven malignancies.

### 2.2 HPV–oral microbiota interactions in special populations

A sequencing analysis among 63 HIV-negative tumor-free MSM demonstrated hrHPV-associated oral microbial shifts only among HIV-negative participants; suggesting that pre-existing HIV-driven oral dysbiosis may mask hrHPV-specific microbial signatures (Escobar-Marcillo et al., 2025). HIV-stratified hrHPV subgroups carried distinct pathogenic bacteria: periodontopathic *Parvimonas* in HIV-negative subjects and pro-inflammatory *Bulleidia* in HIV-infected counterparts, alongside hrHPV-linked perturbations in carbohydrate metabolism and virulence protease pathways (Escobar-Marcillo et al., 2025). This work indicates pre-existing oral dysbiosis driven by HIV masks microbial signatures of hrHPV, and hrHPV further disturbs mucosal microecology by expanding pathogenic taxa.

Higher oral microbial richness among oral-HPV-positive pregnant women was reported, with elevated relative abundance of *Selenomonas*, *Megasphaera* and TM7-related taxa (Tuominen et al., 2018a). Nevertheless, such findings are constrained by the small sample size and exclusive recruitment of pregnant subjects, which limits the generalizability of these conclusions to broader human populations. Separately, infant oral microbiota data from the same group showed oral HPV positivity altered bacterial profiles at genus and family levels without changing overall α-diversity (Tuominen et al., 2018b). HPV carriers and virus-free infants exhibited divergent oral bacterial structures: *Veillonella dispar* prevailed in HPV-negative infants, while *Ureaplasma* and *Propionibacterium* accumulated in HPV-positive ones. As a core infant oral genus, *Veillonella* predicts elevated caries risk later in life (Könönen et al., 1999; Jiang et al., 2016; Mashima et al., 2017). Early HPV exposure disrupts oral commensal balance, chronically impairing mucosal immunity and predisposing individuals to subsequent oral lesions.

Stratified subgroup analyses identified distinct HPV profiles and oral microbial dysbiosis among head and neck squamous cell carcinoma (HNSCC) subtypes (Chan et al., 2022). Non-oral cavity squamous cell carcinoma (SCC) exhibited high HPV prevalence alongside mild microbial disturbance. Conversely, most oral cavity SCC (OSCC) patients were HPV-negative, including numerous triple-negative cases lacking HPV infection, tobacco and alcohol exposure. OSCC was accompanied by substantially severe oral dysbiosis, implying microbiota imbalance may act as an independent oncogenic co-factor (Chan et al., 2022).

### 2.3 HPV and oral microbiota crosstalk in periodontitis

Emerging evidence indicates that periodontitis pathogenesis relies not only on pathogenic bacteria but also on virus-bacterium crosstalk, in which viruses aggravate periodontal lesions via direct cytotoxicity, bacterial synergism and immune modulation. This bidirectional pathogenic loop sustains oral dysbiosis and epithelial barrier injury (Teles et al., 2022). In chronic periodontitis tissues, HPV positivity correlates with enriched *Capnocytophaga ochracea*, which may interact with DNA repair and cytoskeletal proteins (Chowdhry et al., 2019). Patients with HPV-16 infection and deep periodontal pockets exhibit elevated *Porphyromonas* and depleted *Veillonella*, indicating unique microbial signatures in HPV-associated periodontal disease (Shigeishi et al., 2021). A cross-sectional study of 4685 adults found moderate-to-severe periodontitis correlated with oral HPV infection exclusively in females; mediation analysis suggested periodontal inflammation, rather than microbial diversity, independently increases HPV susceptibility (Liang et al., 2026). Furthermore, periodontal pockets create a pro-HPV microenvironment: impaired epithelial barriers facilitate HPV invasion, whereas periodontal pathogens such as *Porphyromonas gingivalis* enhance viral persistence through immune dysregulation, supporting bidirectional pathogenic interactions between periodontal flora and oncogenic HPV (Liang et al., 2026).

### 2.4 HPV-associated oral microbial oncogenic axis

Oral microbial dysbiosis contributes to head and neck carcinogenesis through chronic inflammation, genotoxin release, immune dysregulation and oxidative DNA damage (Constantin et al., 2023). A synergistic tripartite oncogenic axis comprising dysbiotic oral bacteria, hrHPV and *Candida albicans* may promote OSCC progression (Muzio et al., 2021; Constantin et al., 2023; Di Spirito et al., 2023). Periodontal pathogens such as *Fusobacterium*, *Prevotella* and *Campylobacterium* may disrupt epithelial barriers, trigger chronic mucosal inflammation and impair xenobiotic detoxification, potentially facilitating hrHPV adhesion, invasion and genomic integration (Constantin et al., 2023; Di Spirito et al., 2023). Viral E6/E7 oncoproteins degrade p53 and pRb suppressors to drive aberrant cell cycle progression, and microbial virulence factors further amplify HPV-mediated genomic damage (Constantin et al., 2023). As a key fungal co-factor, *C. albicans* secretes candidalysin and carcinogenic acetaldehyde, reinforces polymicrobial biofilm formation, and aggravates local immune dysfunction, creating a feedforward pro-tumor loop together with bacteria and hrHPV (Muzio et al., 2021). This tripartite microbial-viral-fungal crosstalk establishes a self-sustaining oncogenic microenvironment, particularly relevant for OSCC patients without conventional tobacco or alcohol risk exposures.

## 3 Anorectal mucosal microbiota and HPV infection

Owing to specific sexual behaviors, anorectal HPV infection is highly prevalent among MSM (Jacobs et al., 2013; Wei et al., 2021). It is reported that up to 64% of HIV-negative MSM test positive for anal HPV, and HPV is detected in 88.3% of anal cancer (Machalek et al., 2012; Alemany et al., 2015). Nevertheless, how HPV remodels the anorectal microbiome remains incompletely characterized.

In HIV-negative MSM, persistent anal HPV infection associates with elevated *Bacteroides fragilis*, while *Victivallis*, *Negativibacillus*, *Eggerthella* are enriched in infected participants; *Prevotella disiens*, *Gardnerella* and *Negativicoccus* correlate with HPV clearance (Yang et al., 2025). Conversely, *Prevotella disiens*, *Gardnerella* and *Negativicoccus* were enriched in HPV-negative subjects, serving candidate markers for viral clearance (Yang et al., 2025). Cristina et al (Brickman et al., 2024). similarly detected higher *Bacteroides fragilis* levels in MSM diagnosed with HSIL, implying a correlation between this microbe and HPV-mediated lesion advancement. *Bacteroides fragilis* secretes polysaccharide A to regulate CD4^+^ T-cell and inflammatory signaling, which may dampen anorectal antiviral immunity. Enterotoxigenic *B. fragilis* further releases fragilysin to break epithelial barriers and trigger IL-8-driven inflammation, creating a niche conducive to sustained HPV colonization (Sears et al., 2014; Eribo et al., 2022).

Nowak et al. suggested that a higher proportion of MSM with prevalent HPV-16 are associated with a cluster of the anorectal microbiome enriched with *Sneathia* (Nowak et al., 2019). *Sneathia* species produce the adhesin virulence factor FadA, which facilitates mucosal colonization, epithelial invasion and dysregulation of local mucosal immunity (Han et al., 2000; Harwich et al., 2012). Cohort studies focusing on vaginal microbiota have confirmed its correlation with persistent HPV and cervical neoplasia (Lee et al., 2013). It is hypothesized that Sneathia remodels the mucosal microenvironment to impair the host’s capacity to eliminate HPV locally (Nowak et al., 2019).

Co-infection with rectal *Chlamydia trachomatis* or *Neisseria gonorrhoeae* elevates MSM’s hrHPV rate to 70.2% (Ceccarani et al., 2024). Detection of hrHPV exhibited positive correlations with the abundance of *Escherichia* and *Corynebacterium*, whereas negative correlations were identified with the phylum Firmicutes as well as genera *Prevotella*, *Oscillospira* and *Sutterella* (Ceccarani et al., 2024). In HIV-positive MSM, fecal and rectal mucosal microbiota show consistent shifts: *Ruminococcus* accumulates in HSIL patients as a compensatory response to HPV-induced tissue injury, while pathogenic *Pseudomonas aeruginosa* overactivates the kynurenine pathway to aggravate HPV/HIV lesions (Serrano-Villar et al., 2017).

A retrospective microbiome analysis recruited three cohorts of women with high-grade lower-genital-tract lesions, comprising high-risk individuals without anal lesions, patients with anal dysplasia, and subjects with locally advanced anal SCC (Elnaggar et al., 2023). Distinct anorectal microbial community structures were identified across the three groups, while α-diversity exhibited a descending-then-recovering trend from healthy high-risk subjects to dysplasia and cancer patients. LEfSe analysis validated that pro-inflammatory anaerobes including *Peptoniphilus, Prevotella, Porphyromonas* and *Fusobacterium* were markedly enriched in anal cancer specimens. Mechanistically, these taxa trigger sustained mucosal inflammation via TLR-mediated signaling, secrete pro-inflammatory cytokines such as TNF-α and IL-6, and suppress anti-tumor immune responses by recruiting myeloid-derived suppressor cells, thereby creating a permissive microenvironment that may facilitate persistent HPV infection and subsequent anal carcinogenesis (Mudter and Neurath, 2007; Doerflinger et al., 2014; Tett et al., 2021; Elnaggar et al., 2023). These studies suggest a close relationship between changes in microbiota composition, persistent HPV infection, and tumorigenesis. Collectively, these data establish intimate crosstalk between anorectal dysbiosis, sustained HPV infection and anal tumorigenesis. It should be noted that conclusions for the anorectal site are based on a limited number of observational studies, and further research is needed to validate these findings.

## 4 Penile skin-associated microbiota and HPV infection

Penile skin surface is colonized by a broad spectrum of bacterial taxa (Onywera et al., 2020a). Such penile microbial communities are presumed to exert critical impacts on the epidemiological transmission of sexually transmitted infections in male populations. Nevertheless, research exploring the correlations between penile microbiota and genital HPV infection remains insufficient to date.

In HIV-negative men, subclinical HPV infection correlates with penile dysbiosis characterized by anaerobic over-growth and depletion of protective facultative commensals, suggesting penile microbial profiles could regulate HPV susceptibility or viral persistence (Garza-González et al., 2026). Consistently, a cohort study involving 238 heterosexual Black men applied 16S rRNA sequencing to analyze penile-skin microbiota, defining six penile community-state types (CSTs) (Onywera et al., 2020a). The *Corynebacterium*-dominated CST-1 was associated with a reduced prevalence of hrHPV, whereas CST-5 enriched with bacterial vaginosis-associated anaerobes (e.g., *Prevotella*, unclassified Clostridiales, *Porphyromonas*) conferred markedly elevated risks of overall and hrHPV infection. Such anaerobic-dominant microbial signatures are hypothesized to either increase viral vulnerability or delay HPV clearance, though this mechanistic assumption awaits further verification. Multiple independent investigations have also observed enriched penile *Corynebacterium* and diminished HPV infection risk after circumcision (Tobian et al., 2009; Gray et al., 2010; Liu et al., 2013; Zozaya et al., 2016), supporting a protective role of this genus is associated with lower risk of penile HPV; nevertheless, the causal biological pathway linking post-circumcision microbial remodeling to suppressed HPV susceptibility has not yet been clarified.

HIV co-infection acts as a confounding factor that aggravates HPV-mediated penile microbial disturbance, as evidenced by substantially higher α-diversity in HIV/HPV dual-infected men relative to males with isolated HPV or hrHPV infection (Onywera et al., 2020b). Reciprocal crosstalk between HIV and HPV synergistically remodels the penile and broader genital microbial ecosystem. Functionally, HPV-positive men with highly diverse penile microbiota tend to secrete higher local chemokines than those with a *Corynebacterium*-dominated microbiome, and these chemokines may cooperate with Langerhans cells to accelerate HPV elimination (Shannon et al., 2017). Variations in HPV acquisition and clearance efficiency between circumcised and uncircumcised males across distinct penile anatomical regions are largely attributed to disparities in local chemokine concentrations and tissue Langerhans cell distribution (Larke et al., 2011; Shannon et al., 2017). Interpretations for penile microbiota-HPV associations should be treated with caution given the small and heterogeneous body of existing evidence.

## 5 Limitations

This mini-review has several important limitations. First, most evidence for HPV-microbiota relationships across oral, anorectal, and penile sites comes from cross-sectional observational studies, precluding causal or definitive bidirectional inferences. Second, many included studies employ small, highly heterogeneous cohorts with variable demographic features, risk-factor profiles and enrollment criteria. Third, most microbiome data stem from 16S rRNA amplicon sequencing, which suffers from limited species-level taxonomic resolution; moreover, relative-abundance outputs cannot reflect absolute microbial load or total biomass. Fourth, heterogeneity in sampling, DNA extraction and bioinformatic workflows hinders direct cross-study comparisons. Fifth, confounders including smoking, diet, sexual behavior, antibiotic exposure, circumcision, HIV status and other sexually-transmitted co-infections complicate result interpretation. Sixth, evidence is uneven across anatomical sites: oral-site data are comparatively ample, whereas anorectal and penile studies remain sparse and heterogeneous. Findings from oral mucosa should not be simply extrapolated, and current data do not support a unified mechanistic model for all three sites. Further longitudinal cohorts and functional experiments are required to clarify causal mechanisms and validate microbiota-associated biomarkers for non-cervical HPV-related diseases.

## 6 Conclusion

This mini-review provides a narrative synthesis of observed associations and potential bidirectional interactions between HPV and mucosal microbiota in oral, anorectal and penile niches beyond cervicovaginal tissues. Site-specific microbial dysbiosis is associated with impaired mucosal immunity and may facilitate persistent HPV colonization and may contribute to oncogenic progression; meanwhile HPV infection can reshape local microbial composition. Distinct protective and pathogenic bacterial taxa are identified across three anatomical sites. Nevertheless, the supporting evidence is far more robust for the oral cavity, whereas evidence from anorectal and penile mucosa is limited and heterogeneous. Therefore, a uniform mechanistic model for all three anatomical sites cannot be assumed. Future longitudinal cohorts and functional experiments are required to clarify causal mechanisms and develop microecology-based preventive strategies for extragenital HPV-related malignancies.
